# Generalized Edema Caused by Albiglutide: A Case Report

**DOI:** 10.7759/cureus.2801

**Published:** 2018-06-13

**Authors:** Kashif Aziz, Amir Shahbaz, Paria Zarghamravanbakhsh, Syed Ijlal Ahmed, Issac Sachmechi

**Affiliations:** 1 Internal Medicine, Icahn School of Medicine at Mount Sinai Queen Hospital Center, New York, USA; 2 Endocrinology, Icahn School of Medicine at Mount Sinai Queen Hospital Center, New York, USA; 3 Graduate, Liaquat National Hospital and Medical College, Karachi, PAK

**Keywords:** generalized edema, albiglutide, glp-1, type 2 diabetes mellitus, liraglutide

## Abstract

Albiglutide is a long-acting glucagon‐like peptide‐1 (GLP-1) receptor agonist administered by weekly injection. Albiglutide-treated patients experience incidences of adverse effects, including cardiovascular, renal, and gastrointestinal symptoms and injection-site reactions. We report the first case of generalized edema induced by albiglutide. We present a 35-year-old female with type-2 diabetes. She developed generalized edema after the start of albiglutide. Her physical examination and all the laboratory tests, including thyroid function tests, albumin level test, and renal function test were unremarkable, and did not explain her generalized edema. Later on, her albiglutide was discontinued, which lead to the resolution of her edema. We conclude that her generalized edema could be due to a possible side effect of albiglutide.

## Introduction

Albiglutide is a once-weekly glucagon‐like peptide‐1 (GLP-1) agonist. Albiglutide develops through the fusion of two repeats of human GLP-1 molecules to recombinant human albumin. The GLP-1 dimer was used to avoid potential reductions of the interaction of the GLP-1 moiety of the monomer with its receptor in the presence of albumin. A single amino acid replacement (alanine→glycine) renders the molecule resistant to dipeptidyl peptidase-4 inhibitor (DPP-4). The structure of albiglutide provides an extended half-life (∼five days), which may allow weekly or less frequent dosing [1]. Albiglutide improves both fasting and postprandial glucose concentrations in type-2 diabetic patients. Albiglutide, like other GLP-1 inhibitors, improves glucose-dependent glucagon secretion, delays gastric emptying, and suppresses appetite, leading to weight loss. Furthermore, albiglutide improves β-cell function and inhibits β-cell apoptosis in vitro [2]. The most common side effects include gastrointestinal symptoms, a localized reaction at the site of injection, and hypoglycemia [3]. Here, we report the first case of generalized edema induced by albiglutide while the abstract of that case was already presented at a meeting (American Association of Clinical Endocrinologists Annual Congress, 2015) (Sachmechi I, Mahmood A, Shrestha G, Lubana SS: Generalized Edema Caused by Albiglutide, A Case Report, Abstract, The American Association of Clinical Endocrinologists, (2015), https://www.aace.com/files/late-breaking-abstracts-2015.pdf).

## Case presentation

A 35-year-old female with type-2 diabetes mellitus diagnosed five years ago and being managed with metformin was given liraglutide six months ago to improve her glycemic control. However, for a better dosing schedule, liraglutide was subsequently discontinued and a recently improved medication, albiglutide, was added. The following day, post the administration of her first dose, the patient reported a swelling of her lower extremities, which progressed over the next two days to involve the face and upper extremities. She also had a weight gain of five pounds. There was no other possible explanation of generalized edema and the patient did not have cardiac, liver, or renal disease. There were also no recent changes in her medications. Her physical examination was remarkable for generalized edema and periorbital puffiness. There was no stridor and her lungs were clear to auscultation. The further physical examination was unremarkable. Important laboratory investigations are given in Table 1.

**Table 1:** Blood Tests Abbreviations: Thyroid Stimulating Hormone (TSH), Free Thyroxine (FT4), Blood Urea Nitrogen (BUN)

Test	Units	Normal Range	Patient Values
TSH	mIU/ml	0.7-5	3.5
FT4	ng/dl	0.58-1.64	1.1
Creatinine	mg/dl	0.6-1.2	0.9
BUN	mg/dl	7-20	12
Albumin	g/dl	3.5-5.5	4.2

 All the blood tests listed in the above table are normal. Chest X-ray, electrocardiogram (EKG), and ultrasonography of her abdomen were all unremarkable. Due to a cause-and-effect relationship, it was presumed that her generalized edema could be due to a possible side effect of albiglutide. Consequently, albiglutide was discontinued, which led to the resolution of her generalized edema, thereby confirming albiglutide as the causative agent. Liraglutide was then resumed and our patient remained symptom-free.

## Discussion

The adverse effects of albiglutide are mostly gastrointestinal, acute pancreatitis, and hypoglycemia, usually in conjunction with the use of sulfonylureas or insulin and localized skin reactions. Ahren et al. performed an integrated safety analysis of  albiglutide compared with a placebo and/or active comparators (a dipeptidyl peptidase-4 inhibitor, insulin, sulphonylurea, and thiazolidinedione) [4]. In the integrated analysis of seven phase three clinical trials, albiglutide-treated patients experienced frequencies of adverse events, including cardiovascular and renal, similar to the all-comparators group treated with other type-2 diabetes mellitus (T2DM) medications or placebos. Albiglutide treatment was associated with higher rates of diarrhea and injection-site reactions, but not increased nausea and vomiting, versus all comparators [4]. Rendell discussed the safety of albiglutide for the treatment of type-2 diabetes mellitus and explained that the gastrointestinal side effects of albiglutide are not much greater than other antidiabetic drugs. It has been studied and appears safe at all stages of renal failure [5]. Fisher et al. did a meta-analysis on the cardiovascular safety of albiglutide. They concluded that cardiovascular events were not significantly more likely to occur with albiglutide than with all comparators (glimepiride, insulin glargine, insulin lispro, liraglutide, pioglitazone, or sitagliptin) [6]. As we discussed different clinical trials and studies above, we could not find a single piece of evidence from the literature regarding the association of generalized edema with albiglutide. To the best of our knowledge, this is the first case report of generalized edema caused by albiglutide. The pathophysiology is not clear. Since the patient's albumin level was normal and changes in oncotic pressure cannot be the cause, the most likely possible mechanism was fluid retention.

## Conclusions

This case highlights the potential for generalized edema as an adverse effect of albiglutide. Patients prescribed albiglutide should be aware of such adverse events. If lower extremity swellings occur with its use, the medication should be discontinued to prevent the progression of generalized edema.
